# Relationships between high-sensitive C-reactive protein and markers of arterial stiffness in hypertensive patients. Differences by sex

**DOI:** 10.1186/1471-2261-12-37

**Published:** 2012-06-07

**Authors:** Manuel A Gomez-Marcos, Jose I Recio-Rodríguez, Maria C Patino-Alonso, Cristina Agudo-Conde, Leticia Gomez-Sanchez, Emiliano Rodriguez-Sanchez, Marta Gomez-Sanchez, Vicente Martinez-Vizcaino, Luis Garcia-Ortiz

**Affiliations:** 1 Primary Care Research Unit, La Alamedilla Health Center, Avda. Comuneros 27, Salamanca, 37003, Spain; 2 Social and Health Care Research Center, University of Castilla-La Mancha, Calle Altagracia, 50, Cuenca, 19071, Spain

**Keywords:** Hypertension, High-sensitive C-reactive protein, Arterial stiffness

## Abstract

**Background:**

The present study was designed to evaluate the relationship between high-sensitivity C-reactive protein (hs-CRP) and arterial stiffness according to sex in patients with arterial hypertension.

**Methods:**

A case-series study was carried out in 258 hypertensive patients without antecedents of cardiovascular disease or diabetes mellitus. Nephelometry was used to determine hs-CRP. Office or clinical and home blood pressures were measured with a validated OMRON model M10 sphygmomanometer. Ambulatory blood pressure monitoring was performed with the SpaceLabs 90207 system. Pulse wave velocity (PWV) and central and peripheral augmentation index (AIx) were measured with the SphygmoCor system, and a Sonosite Micromax ultrasound unit was used for automatic measurements of carotid intima-media thickness (IMT). Ambulatory arterial stiffness index and home arterial stiffness index were calculated as “1-slope” from the within-person regression analysis of diastolic-on-systolic ambulatory blood pressure.

**Results:**

Central and peripheral AIx were greater in women than in men: 35.31 ± 9.95 vs 26.59 ± 11.45 and 102.06 ± 20.47 vs 85.97 ± 19.13, respectively. IMT was greater in men (0.73 ± 0.13 vs 0.69 ± 0.10). hs-CRP was positively correlated to IMT (r = 0.261), maximum (r = 0.290) and to peripheral AIx (r = 0.166) in men, and to PWV in both men (r = 0.280) and women (r = 0.250). In women, hs-CRP was negatively correlated to central AIx (r = −0.222). For each unit increase in hs-CRP, carotid IMT would increase 0.05 mm in men, and PWV would increase 0.07 m/sec in men and 0.08 m/sec in women, while central AIx would decrease 2.5 units in women. In the multiple linear regression analysis, hs-CRP explained 10.2% and 6.7% of PWV variability in women and men, respectively, 8.4% of carotid IMT variability in men, and 4.9% of central AIx variability in women.

**Conclusions:**

After adjusting for age, other cardiovascular risk factors and the use of antihypertensive and lipid-lowering drugs, hs-CRP was seen to be positively correlated to carotid IMT in men, and negatively correlated to central AIx in women. The association of hs-CRP to arterial stiffness parameters differs between men and women.

## Background

The association of high-sensitivity CRP (hs-CRP) to cardiovascular morbidity-mortality has not been sufficiently clarified. Some studies have identified hs-CRP as an independent predictor of cardiovascular events, hypertension and diabetes [1-3]. It offers prognostic information additional to that of the classical factors used to estimate cardiovascular risk, and reclassifies a substantial proportion of “intermediate risk” individuals as presenting high risk [3,4]. Kaptoge et al. [5], in a metaanalysis of 160,309 patients without antecedents of cardiovascular disease, concluded that hs-CRP concentration shows a continuous association to the risk of coronary disease, cerebral thrombosis and cardiovascular mortality. Nevertheless, this association is conditioned by the conventional cardiovascular risk factors. However, its role in the pathogenesis of arteriosclerosis has not been demonstrated [1,6-8].

The studies that analyze the relationship between hs-CRP and arterial stiffness, as well as behavior according to sex, are inconclusive [9-11]. A prospective study in males subjected to 20 years of follow-up has concluded that pulse wave velocity (PWV) is related to cumulative exposure to hs-CRP, while the augmentation index (AIx) is more related to the values present at the time of analysis [12]. Regarding the relationship between hs-CRP and intima-media thickness (IMT), a number of studies have reported a positive correlation, particularly in hypertensive elderly individuals [8,13], while another study has only found an association in women [14]. The association of the different arterial stiffness measures to hs-CRP and its behavior according to sex in hypertensive individuals have not been studied to date. The present study was designed to evaluate the relationship between hs-CRP and arterial stiffness as evaluated by carotid IMT, PWV, central and peripheral AIx, ambulatory arterial stiffness index (AASI) and home arterial stiffness index (HASI) according to sex in patients with primary arterial hypertension.

## Methods

### Study design and population

A cross-sectional study was carried out in a primary care setting. We consecutively included all hypertensive subjects visiting primary care clinics between January 2008 and January 2011, and referred to the research unit for the assessment of cardiovascular risk. Hypertension was diagnosed when the mean of three recordings in the clinic under basal conditions and separated in time revealed systolic blood pressure (SBP) ≥140 and/or diastolic blood pressure (DBP) ≥ 90 mmHg. On each visit at least two recordings were made, spaced more than one minute apart. The included 258 hypertensive patients were aged 30–80 years without history of cardiovascular disease (ischemic heart disease or stroke) or diabetes mellitus. Sample size calculation indicated that the 258 patients included in the study were sufficient to detect a minimum correlation coefficient between hs-CRP and arterial stiffness parameters of 0.20 in a two-sided test, with a significance level of 0.05 and a power of 0.90. The study was approved by an independent ethics committee of Salamanca University Hospital (Spain), and all participants gave written informed consent according to the general recommendations of the Declaration of Helsinki [15].

### Measurement

The clinical, anthropometric and analytical data collected are shown in Table 1. The measurement procedure has been described previously [16]. Hs-CRP was determined by the nephelometric method (Beckman Instrument APS; Beckman Coulter Inc., Fullerton, CA, USA) [17].

**Table 1:** Characteristics of study patients, overall and by sex

**Variable**	**Overall (n = 258)**	**Males (n = 153)**	**Females (n = 105)**	**P value**
Age (years)	53.27 ± 12.01	52.18 ± 12.20	54,85 ± 11.60	0.080
Serum glucose (mg/dl)	87.69 ± 10.80	88.16 ± 11.53	86.99 ± 9.61	0.398
Glycated hemoglobin (%)	5.05 ± 0.54	5.06 ± 0.58	5.02 ± 0.48	0.566
**Office BP (mmHg)**				
SBP	139.93 ± 16.75	141.35 ± 15.90	137.84 ± 17.81	0.099
DBP	88.34 ± 10.69	88.33 ± 10.53	88.37 ± 10.98	0.975
Pulse pressure	52.23 ± 12.82	53.61 ± 12.70	50.20 ± 12.79	0.036
Heart rate	72.37 ± 12.89	70.09 ± 12.62	75.74 ± 12.60	0.001
**ABPM 24 hours (mmHg)**				
SBP	126.71 ± 12.83	127.66 ± 12.20	125.31 ± 13.63	0.149
DBP	78.72 ± 9.86	80.13 ± 9.58	76.65 ± 9.93	0.005
Pulse pressure	47.99 ± 9.50	47.53 ± 8.53	48.66 ± 10.76	0.348
Heart rate	71.92 ± 10.62	71.03 ± 11.47	73.24 ± 9.11	0.103
**Home BP (mmHg)**				
SBP	127.23 ± 14.49	128.69 ± 13.55	125.11 ± 15.58	0.051
DBP	81.62 ± 9.85	81.92 ± 9.45	81.19 ± 10.43	0.557
Pulse pressure	45.60 ± 10.07	46.77 ± 9.01	43.91 ± 11.26	0.026
Heart rate	68.62 ± 9.17	67.35 ± 8.98	70.45 ± 9.18	0.007
Current smokers (%)	64 (24,8)	46 (30.1)	18 (17.1)	0.018
Obesity (%) *	70 (27,1)	45 (29.4)	25 (23.8)	0.320
BMI (kg/m^2^)	27.92 ± 3.83	28.30 ± 3.20	27.37 ± 4.55	0.054
Waist circumference (cm)	95.60 ± 11.12	99.45 ± 9.14	90.03 ± 11.41	p < 0.001
Dyslipidemia (%)	206 (79,8)	121 (79.1)	85 (81.0)	0.633
Total cholesterol (mg/dL)	208.96 ± 36.94	205.37 ± 35.13	214.27 ± 39.02	0.059
Triglycerides (mg/dL)	126.34 ± 73.35	135.95 ± 85.98	112.17 ± 45.96	0.011
LDL-cholesterol (mg/dL)	130.64 ± 33.00	129.59 ± 30.35	132.17 ± 36.62	0.546
HDL-cholesterol (mg/dL)	53.28 ± 13.01	48.90 ± 10.94	59.73 ± 13.16	p < 0.001
No HDL-cholesterol (mg/dL)	155,75 ± 36.48	156.50 ± 35.41	154.64 ± 38.15	0.692
Atherogénic Index	4,12 ± 1.13	4.39 ± 1.19	3.73 ± 0.92	p < 0.001
Cardiovascular risk DAgostino	15.29 ± 12.52	19.19 ± 14.15	9.55 ± 6.19	p < 0.001
hs-CRP (mg/L)	2.73 ± 4.02	2.94 ± 4.81	2.41 ± 2.37	0.310
Fibrinogen, g/L	317.05 ± 61.39	314.57 ± 63.55	320.69 ± 58.21	0.441
Maximum IMT (mm)	0.88 ± 0.14	0.90 ± 0.15	0.86 ± 0.11	0.012
Mean IMT (mm)	0.71 ± 0.12	0.73 ± 0.13	0.69 ± 0.10	0.011
PWV (m/sec)	8.65 ± 2.06	8.66 ± 2.14	8.64 ± 1.96	0.952
CAIx	30.12 ± 11.66	26.59 ± 11.45	35.31 ± 9.95	p < 0.001
PAIx	92.52 ± 21.19	85.97 ± 19.13	102.06 ± 20.47	p < 0.001
AASI	0.37 ± 0.06	0.37 ± 0.06	0.38 ± 0.06	0.050
AASI-BPVR	0.16 ± 0.16	0.15 ± 0.17	0.18 ± 0.13	0.101
Awake-AASI	0.37 ± 0.06	0.37 ± 0.05	0.38 ± 0.06	0.061
Sleep-AASI	0.38 ± 0.15	0.38 ± 0.14	0.39 ± 0.16	0.534
HASI	0.59 ± 0.18	0.58 ± 0.20	0.61 ± 0.15	0.365
HASI-BPVR	0.29 ± 0.24	0.25 ± 0.26	0.35 ± 0.19	0.001
Antihypertensive Drugs (%)	101 (39.1)	61 (39.9)	40 (38.1)	0.774
Lipid-lowering Drugs (%)	48 (18.6)	31 (20.3)	17 (16.2)	0.409

Data for qualitative variables are expressed as n (%) and quantitative variables as mean ± standard deviation.

* Obesity: *BMI* ≥ 30 Kg/m²or Waist circumference ≥ 88 cm in men and ≥ 102 cm in women. *SBP*: Systolic blood pressure; *DBP*: Diastolic blood pressure; *AMBP*: Ambulatory blood pressure monitoring; *BMI*, body mass index; *hs-CRP*: high-sensitive C-reactive protein; *HDL*, high density lipoprotein; LDL, lowdensity lipoprotein; *IMT*: Intima Media Thickness; *PWV*: pulse wave velocity; *CAIx*: Central augmentation index; *PAIx*: Peripheral augmentation index, *AASI*: Ambulatory arterial stiffness index, *BPVR*: Blood pressure variability ratio; *HASI*: Home arterial stiffness index.

### Blood pressure

#### Office or clinical blood pressure

Measurement was obtained by performing three measurements of systolic (SBP) and diastolic blood pressure (DBP), using the average of the last two, with a validated OMRON model M10 sphygmomanometer (Omron Health Care, Kyoto, Japan), and following the recommendations of the European Society of Hypertension [18]. The mean of the last two measurements obtained by the nurse of the research unit from the arm with high blood pressure was used for the study.

#### Home blood pressure (HBP)

Three measurements were made in the morning (between 6:00 and 9:00 a.m.), and three in the afternoon/evening (between 6:00 and 9:00 p.m.), over a period of 7 days, with a minimum interval of one minute between measurements, and excluding the first measurement and the values corresponding to the first day of measurement [19]. The same sphygmomanometer model used to measure blood pressure in the office was employed.

#### Ambulatory blood pressure monitoring (ABPM)

ABPM was performed on a day of standard activity, with an adequate cuff for the size of the patient’s arm. A control system (Spacelabs 90207, Healthcare, Issaquah, Washington, USA), validated according to the protocol of the British Hypertension Society, was used [20]. The records of readings considered to be valid were ≥ 80% of the total. The monitor was programmed for obtaining blood pressure measurements every 20 min during the waking period and every 30 min during the resting period. Individual correction was made of the waking and sleeping hours reported by the patient.

### Vascular assessment

#### Assessment of carotid intima-media thickness (IMT)

Carotid ultrasound to assess IMT was performed by two investigators trained for this purpose before starting the study. The reliability of assessment was evaluated before the study, using the intraclass correlation coefficient, which showed values of 0.974 (95%CI: 0.935 to 0.990) for intra-observer agreement on repeated measurements in 20 subjects, and 0.897 (95%CI: 0.740 to 0.959) for inter-observer agreement. According to the Bland-Altman analysis, the limit of inter-observer agreement was 0.022 (95%CI: -0.053 to 0.098), and the limit of intra-observer agreement was 0.012 (95%CI: -0.034 to 0.059). A Sonosite Micromax ultrasound device paired with a 5–10 MHz multifrequency high-resolution linear transducer with Sonocal software was used for performing automatic measurements of CC-IMT, obtained automatically 120 values, 10 measurements in each of the 12 projections, in order to optimize reproducibility. Measurements were made of the common carotid artery after the examination of a longitudinal section of 10 mm at a distance of 1 cm from the bifurcation, performing measurements in the near wall, and in the far wall in the lateral, anterior and posterior projections, following an axis perpendicular to the artery to discriminate two lines - one for the intima-blood interface and the other for the media-adventitia interface. Using mean average values and maximum average values calculated automatically by the software. The measurements were obtained with the subject lying down, with the head extended and slightly turned opposite to the exploratory side, following the recommendations of the Manheim Carotid Intima-Media Thickness Consensus [21].

#### Pulse wave velocity (PWV) and peripheral (PAIx) and central (CAIx) augmentation index

Were estimated through the SphymgoCor System (AtCor Medical Pty Ltd Head Office, West Ryde, Australia). Using the SphygmoCor System (Px Pulse Wave Analysis) by an investigator, with the patient in the sitting position and resting the arm on a rigid surface, pulse wave analysis was made with a sensor in the radial artery, using mathematical transformation to estimate the aortic pulse wave. The reliability of which was evaluated before the study began using the CAIx intra-class correlation coefficient, which showed values of 0.974 (95%CI: 0.936 to 0.989) for intra-observer agreement on repeated measurements in 22 subjects and according to the Bland-Altman analysis the limits of intra-observer agreement was 0.454 (95%CI:−9.876 to 10.785). From the morphology of the aortic wave, Central AIx was estimated using the following formula: Increase in central pressure × 100/pulse pressure. Peripheral AIx was calculated as follows: (second peak systolic blood pressure [SBP2] − diastolic blood pressure [DBP])/(first peak SBP-DBP) × 100 (%). Using the SphygmoCor System (Vx pulse wave velocity), and with the patient in the supine position, the pulse wave of the carotid and femoral arteries was analyzed, estimating the delay with respect to the ECG wave and calculating the PWV. Distance measurements were taken with a measuring tape from the sternal notch to the carotid and femoral arteries at the sensor location.

### Ambulatory arterial stiffness index (AASI) and home arterial stiffness index (HASI)

For AASI and HASI estimation, the regression slope of diastolic on systolic blood pressure was computed for each individual on the basis of 24-hour ABPM (AASI) and also HBP readings (HASI) over 6 days. AASI as well as HASI were defined as one minus the respective regression slope of DBP on SBP. AASI was also computed from waking or sleeping blood pressure. Blood pressure variability ratio (BPVR) was defined as SD (SBP)/SD(DBP), AASI (BPVR) as 1-[1/SD (SBP)/SD (DBP)] in 24-hour blood pressure [22-24], and HASI (BPVR) as 1-[1/SD (SBP)/SD (DBP)] over 7 days of HBP recording.

The individuals performing the different tests were blinded to the clinical data of the patient. All organ damage assessment measures were made within a period of 10 days.

### Statistical analysis

Continuous variables were expressed as the mean ± standard deviation (SD), while frequency distributions were used for qualitative variables. The difference in means between two-category quantitative variables has been analyzed using the Student *t*-test for independent samples and Ji Square for qualitative variables. Pearson’s correlation coefficient was used to estimate the relationship between quantitative variables. We performed linear regression analysis using high-sensitive C-reactive protein as independent variables and using IMT, PWV and CAIx as dependent variables. We have performed multiple linear regression analysis with interaction effects (between gender and hs-CRP) using IMT, PWV and CAIx as dependent variables and high-sensitive C-reactive protein as independent variables, establishing 7 models: model 1, without adjusted; model 2, adjusting for gender and interaction gender x hs-CRP, model 3, adding age; model 4, adding the waist circumference; model 5, adding 24 hours SBP and 24 hours heart rate; model 6, adding Atherogenic Index (Atherogenic Index = Total cholesterol/HDL-cholesterol); model 7, adding Antihypertensive and Lipid-lowering Drugs. We performed multiple linear regression analysis using IMT, PWV and CAIx as dependent variables and high-sensitive C-reactive protein as independent variables, establishing 6 models: model 1, without adjusted; model 2, adjusting for age; model 3, adding the waist circumference; model 4, adding SBP, ABPM 24 hours and heart rate 24 hours; model 5, adding Atherogenic Index; model 6, adding Antihypertensive and Lipid-lowering Drugs. All analyzes were performed by gender and variables not normally distributed we used the neperian logarithm (LN). The data were analyzed using the SPSS version 18.0 statistical package (SPSS Inc., Chicago, Illinois, USA).

## Results

The clinical characteristics, cardiovascular risk factors, blood pressure values, IMT, PWV, CAIx, PAIx, AASI, HASI and antihypertensive and lipid-lowering drugs considered globally and by sexes are reported in Table 1. Women showed higher central and peripheral AIx values and home arterial stiffness index-blood pressure variability ratio (HASI-BPVR) values, while men showed greater cardiovascular risk and common carotid IMT values, without differences in hs-CRP.

Table 2 reflects the correlation among hs-CRP, risk factors and IMT, PWV, CAIx, PAIx, AASI, HASI considered globally and according to sexes. Ambulatory systolic (SBP) and diastolic blood pressure (DBP), cardiovascular risk estimated from the Framingham equation, mean and minimum IMT, and peripheral AIx (PAIx) were positively correlated to hs-CRP in men. In women, HDL-cholesterol and central AIx (CAIx) were negatively correlated, while the atherogenic index and ambulatory arterial stiffness index (AASI) (24 hours and resting) were positively correlated to hs-CRP. Waist circumference and PWV showed positive correlations in both sexes.

**Table 2:** Correlation of arterial stiffness measurement and high-sensitive C-reactive protei**n**

**Variable**	**Overall (n = 258)**	**Males (n = 153)**	**Females (n = 105)**
**Office BP (mmHg**)			
SBP	0.048	0.038	0.054
DBP	0.040	0.045	0.031
Pulse pressure	0.026	−0.004	0.060
Heart rate	0.186**	0.206*	0.189
**ABPM 24 hours (mmHg)**			
SBP	0.126*	0.196*	0.023
DBP	0.055	0.161*	−0.125
Pulse pressure	0.115	0.100	0.145
Heart rate	0.246**	0.306**	0.144
**Home BP (mmHg)**			
SBP	0.185**	0.212**	0.138
DBP	0.137*	0.188*	0.047
PP	0.134*	0.123	0.142
Heart rate	0.189**	0.200*	0.196
Total cholesterol (mg/dL)	−0.004	0.044	−0.060
LDL-cholesterol (mg/dL)	0.063	0.094	0.031
HDL-cholesterol (mg/dL)	−0.198**	−0.138	−0.282**
No HDL-cholesterol (mg/dL)	0.063	0.083	0.034
Atherogenic Index	0.171**	0.141	0.211*
Ln Cardiovascular risk DAgostino	0.188**	0.238**	0.086
Waist circumference (cm)	0.235**	0.215**	0.275**
Maximum IMT (mm)	0.194**	0.290**	0.011
Mean IMT (mm)	0.174**	0.261**	0.010
PWV (m/sec)	0.280**	0.250**	0.319**
CAIx	−0.048	0.067	−0.222*
PAIx	0.068	0.166*	−0.058
AASI	0.067	−0.027	0.215*
AASI-BPVR	−0.012	0.012	−0.049
Awake-AASI	0.057	−0.034	0.198
Sleep-AASI	0.093	0.005	0.240*
HASI	−0.013	−0.044	0.057
HASI-BPVR	−0.049	−0.059	0.004

*BP*: blood pressure; *SBP*: Systolic blood pressure; *DBP*: Diastolic blood pressure; *AMBP*: Ambulatory blood pressure monitoring; *HDL*, high density lipoprotein; *LDL*, low density lipoprotein; *IMT*: Intima Media Thickness; *PWV*: pulse wave velocity; *CAIx*: Central augmentation index; *PAIx*: Peripheral augmentation index, *AASI*: Ambulatory arterial stiffness index, *BPVR*: Blood pressure variability ratio; *HASI*: Home arterial stiffness index.

**Figure 1:**
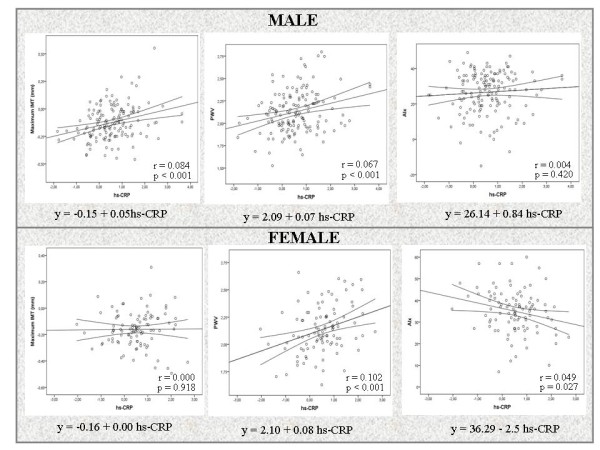
Simple linear regression lines, regression equations and r and P values showing the correlations between high-sensitive C-reactive protein and pulse wave velocity, intima media thickness and central augmentation index.

Figure 1 shows the simple linear regression straight lines of hs-CRP as independent variable and IMT maximum, PWV and AIx as dependent variables. For each unit increase in hs-CRP, carotid IMT would increase 0.05 mm in men, with practically no changes in women (β 0.002, p = 0.918), while PWV would increase 0.07 m/sec in men and 0.08 m/sec in women. Lastly, for each unit increase in hs-CRP, the CAIx would decrease 2.5 units in women.

The interaction analysis, showed that the effect of hs-CRP toward IMT maximum and CAIx depends of the gender (P < 0.05) (Table 3).

**Table 3:** Regression models with the high-sensitive C-reactive protein adjusted for each measure of arterial stiffness, with interaction effect

		**Overall (n = 258)**			
**Variable**	**β**	**P Value**	**95%CI**	**R**^**2**^	**Adjusted R**^**2**^
**Dependent variable:**					
**IMT maximun**					
Model 1	0.031	0.002	0.011 to 0.052	0.037	
Model 2	0.002	0.923	−0.030 to 0.034		0.060
Model 3	−0.009	0.432	−0.033 to 0.014		0.491
Model 4	−0.011	0.368	−0.035 to 0.013		0.486
Model 5	−0.007	0.549	−0.031 to 0.016		0.516
Model 6	−0.010	0.413	−0.033 to 0.014		0.527
Model 7	−0.008	0.502	−0.032 to 0.016		0.525
**Dependent variable:**					
**PWV**					
Model 1	0.070	0.000	0.040 to 0.100	0.078	
Model 2	0.070	0.000	0.040 to 0.101		0.071
Model 3	0.050	0.000	0.025 to 0.075		0.371
Model 4	0.043	0.001	0.018 to 0.069		0.377
Model 5	0.028	0.035	0.002 to 0.054		0.432
Model 6	0.027	0.043	0.001 to 0.053		0.442
Model 7	0.027	0.046	0.000 to 0.053		0.440
**Dependent variable:**					
**CAIx**					
Model 1	−0.617	0.455	−2.241 to 1.006	0.002	
Model 2	−2.511	0.044	−4.956 to −0.066		0.131
Model 3	−2.983	0.010	−5.253 to −0.713		0.254
Model 4	−2.587	0.025	−4.845 to −0.329		0.247
Model 5	−2.420	0.032	−4.626 to −0.214		0.315
Model 6	−2.453	0.031	−4.675 to −0.231		0.311
Model 7	−2.711	0.017	−4.937 to −0.486		0.322

*Model 1*: C-reactive protein.

*Model 2*: Adjusted by Gender and interaction gender x hs-CRP. Gender (coded 1 for male and 0 for female).

*Model 3*: Adjusted bay Model 2 and age.

*Model 4*: Adjusted by Model 3 and waist circumference.

*Model 5*: Adjusted by Model 4 and 24 hours systolic blood pressure (SBP) and 24 hours Heart rate.

*Model 6*: Adjusted by Model 5 and atherogenic Index.

*Model 7*: Adjusted by Model 6 and Antihypertensive and Lipid-lowering Drugs.

Interaction gender x hs-CRP IMT maximum: Model 2 (p = 0.023); Model 3 (p = 0.011); Model 4 (p = 0.010) Model 5 (p = 0.012); Model 6 (p = 0.009); Model 7 (p = 0.013).

Interaction gender x hs-CRP CAIx: Model 2 (p = 0.035); Model 3 (p = 0.042); Model 4 (p = 0.050); Model 5 (p = 0.019); Model 6 (p = 0.020); Model 7 (p = 0.015).

In the *PWV*, the interactions gender x hs-CRP, were not statistically significant.

The multiple linear regression models showed that in males, 8.4% (β = 0.049) of the variability of IMT could be explained by hs-CRP – statistical significance being maintained after the adjustments made in the different models (R^2^ = 0.545, beta 0.026 and p = 0.016). In men, 6.7% of the variability of PWV could be explained by hs-CRP (beta =0.066), versus 10.2% in women (beta = 0.078). Statistical significance was maintained after adjusting for age in men (beta = 0.040; p = 0.019), but was lost in the next model with waist circumference. In contrast, in women statistical significance was maintained to adjustment of the fourth model (age, waist circumference, 24-hour SBP and heart rate) (β = 0.043, p = 0.030). In women, 4.9% of the variability of CAIx could be explained by hs-CRP, statistical significance being maintained after adjusting of the different models (β = −3.134, p = 0.009). The association of hs-CRP to the rest of the parameters used to assess arterial stiffness disappeared after adjusting for age (Table 4).

**Table 4:** Regression models with the high-sensitive C-reactive protein adjusted for each measure of arterial stiffness

	**Males (n = 153)**				**Females (n = 105)**			
**Variable**	**β**	**Sig.**	**95%CI**	**AdjustedR**^**2**^	**β**	**Sig.**	**95%CI**	**AdjustedR**^**2**^
**Dependent variable: IMT maximun**								
Model 1	0.049	0.000	0.022 to 0.075	0.084	0.002	0.918	−0.028 to 0.032	0.000
Model 2	0.029	0.004	0.009 to 0.048	0.511	−0.009	0.452	−0.031 to 0.014	0.430
Model 3	0.025	0.013	0.005 to 0.045	0.508	−0.007	0.546	−0.031 to 0.016	0.425
Model 4	0.027	0.011	0.006 to 0.048	0.537	−0.003	0.827	−0.026 to 0.021	0.456
Model 5	0.026	0.013	0.006 to 0.047	0.549	−0.006	0.647	−0.030 to 0.018	0.459
Model 6	0.026	0.016	0.005 to 0.046	0.545	−0.003	0.782	−0.028 to 0.021	0.454
**Dependent variable:**	**PWV**							
Model 1	0.066	0.001	0.026 to 0.106	0.067	0.078	0.001	0.031 to 0.124	0.102
Model 2	0.040	0.019	0.007 to 0.074	0.370	0.064	0.001	0.025 to 0.103	0.372
Model 3	0.032	0.064	−0.002 to 0.066	0.381	0.060	0.004	0.019 to 0.100	0.371
Model 4	0.020	0.254	−0.015 to 0.056	0.412	0.043	0.030	0.004 to 0.081	0.482
Model 5	0.021	0.233	−0.014 to 0.056	0.420	0.034	0.086	−0.005 to 0.072	0.504
Model 6	0.018	0.308	−0.017 to 0.053	0.427	0.029	0.151	−0.011 to 0.070	0.497
**Dependent variable:**	**CAIx**							
Model 1	0.839	0.420	−1.211 to 2.889	0.004	−2.511	0.027	−4.729 to −0.293	0.049
Model 2	−0.182	0.847	−2.047 to 1.682	0.197	−2.799	0.012	−4.967 to −0.630	0.092
Model 3	0.020	0.983	−1.837 to 1.877	0.172	−2.476	0.031	−4.718 to −0.235	0.094
Model 4	1.160	0.218	−0.692 to 3.011	0.279	−2.689	0.019	−4.929 to −0.449	0.174
Model 5	1.135	0.233	−0.737 to 3.008	0.273	−2.745	0.019	−5.026 to −0.464	0.172
Model 6	1.130	0.238	−0.757 to 3.017	0.270	−3.134	0.009	−5.456 to −0.812	0.204

*Model 1*: C-reactive protein.

*Model 2*: Adjusted for Age.

*Model 3*: Adjusted for model 2 and Waist circumference.

*Model 4*: Adjusted for model 3 and 24 hours Systolic blood pressure and 24 hours Heart rate.

*Model 5*: Adjusted for model 4 and atherogenic Index.

*Model 6*: Adjusted for model 5 and Antihypertensive and Lipid-lowering Drugs.

## Discussion

The present study shows that the correlation of hs-CRP to the IMT, PWV, CAIx, PAIx, AASI, HASI in hypertensive patients behaves differently in men and women. In men, a positive correlation to IMT is observed, while in women a negative correlation to CAIx and a positive correlation to AASI is noted. PWV shows a positive correlation in both sexes. After adjusting for age, waist circumference, 24-hour heart rate and SBP, atherogenic index and the use of antihypertensive and lipid-lowering drugs, the correlation of hs-CRP to carotid IMT in men and to CAIx in women was seen to be maintained. The association to PWV was maintained in men only on adjusting for age, while in women the correlation was maintained with waist circumference, SBP and 24-hour heart rate, after adjusting for age.

In coincidence with the data published by Kaptoge [5], the hs-CRP concentration was higher in males, though CAIx was found to be higher in women than in men, in agreement with other authors [11,25]. The global analysis of the sample revealed correlations between hs-CRP and the different risk factors similar to those published by other investigators [5].

In coincidence with the findings of other studies, mean IMT was greater in men than in women [26]. The association between IMT and hs-CRP only proved significant in men – and this association was maintained after adjusting for the different models used. The studies that have examined the relationship between hs-CRP and carotid IMT in the general population have yielded contradictory results. In this sense, not all of them have confirmed that an increase in hs-CRP implies an increase in IMT [27-30]. In this same line, in hypertensive patients some authors have reported a positive association to the development and progression of carotid atherosclerosis [8,31,32], even in cases of recently diagnosed hypertension [33]. However, Choi et al. [34] observed no association between hs-CRP and carotid atherosclerosis in either hypertensive patients or in normotensive individuals. Likewise, the behavior according to patient sex is not clear. In effect, while Makita et al. [27] concluded that in the general population hs-CRP may serve as a surrogate marker for atheroma plaque formation in men but not in women, Sanders et al. [26] found IMT to be significantly associated to hs-CRP in women but not in men, after adjusting for risk factors – the conclusion being that the relationship between hs-CRP and the progression of early carotid atherosclerosis shows gender differences.

Carmel et al. [12], in a prospective study of the association between PWV and hs-CRP, recorded a positive association similar to that seen in the present study, though in this case the behavior between sexes was found to be different after adjustment in the multiple regression analysis. This suggests that inflammation plays a role in aortic stiffness as assessed with PWV, but that patient sex could exert an influence.

In our study, hs-CRP was correlated negative with CAIx in Woman (r = −0.222), though Janner et al., in the general population, only observed a relationship in young males [11]. These data coincide with the findings of Carmel et al. [12] in men, where after adjusting in multivariate models only fibrinogen remained as a weak predictor between CAIx and hs-CRP. This relationship, could be explained, by CAIx values are higher in women and decrease with increasing BMI. The BMI may be a confounding factor in this negative correlation of hs-CRP with CAIx.

On the other hand, the disparity of results relating hs-CRP to PWV and CAIx in males reinforces the idea that the findings are not inter-exchangeable and differ according to patient sex [35].

The present study has some limitations, including its cross-sectional design, which does not allow us to establish cause-effect relationships between hs-CRP and the arterial stiffness parameters. In turn, the study population was recruited through consecutive sampling, which precludes generalization of the results. Nevertheless, the distribution of the population analyzed is similar to that of the real-life population of hypertensive subjects with cardiovascular risk factors and no previous cardiovascular disease. Lastly, estimation of the central PWV based on the radial PWV, using the transference function, has been questioned [36-38].

## Conclusions

In conclusion, this study shows that after adjusting for age, cardiovascular risk factors and the use of antihypertensive and lipid-lowering drugs, hs-CRP exhibits a positive correlation to IMT in men, a negative correlation to CAIx in women, and a positive correlation to PWV in both sexes. Therefore, in order to clarify the role of hs-CRP in relation to the parameters commonly used to assess arterials stiffness in both males and females, prospective studies must be carried out to clarify this association and to define the differences between sexes.

## Abbreviations

hs-CRP: High-sensitive CRP; PWV: Pulse wave velocity; AIx: Augmentation index; IMT: Intima-media thickness; AASI: Ambulatory arterial stiffness index; HASI: Home arterial stiffness index; SBP: Systolic blood pressure; DBP: Diastolic blood pressure; HBP: Home blood pressure; ABPM: Ambulatory blood pressure monitoring; CAI: Central augmentation index; PAI: Peripherical augmentation index; BPVR: Blood pressure variability ratio.

## Competing interests

The authors declare that they have no competing interests associated with this paper.

## Authors’ contributions

MAGM devised the study, designed the protocol, participated in fund raising, interpretation of results, prepared the manuscript draft and corrected the final version of the manuscript. JIRR and CAC participated in the study design, data collection and manuscript review. MCPA and VMV performed all analytical methods, interpretation of results, and manuscript review. ERS, LGS and MGS participated in the study design, interpretation of results, and manuscript review. LGO participated in the protocol design, fund raising, analysis of results, and final review of the manuscript. Finally, all authors reviewed and approved the final version of the manuscript.

## Pre-publication history

The pre-publication history for this paper can be accessed here:

http://www.biomedcentral.com/1471-2261/12/37/prepub
